# Do environmental risk factors for the development of psychosis distribute differently across dimensionally assessed psychotic experiences?

**DOI:** 10.1038/s41398-021-01265-2

**Published:** 2021-04-19

**Authors:** Jan Cosgrave, Ross J. Purple, Ross Haines, Kate Porcheret, Dalena van Heugten-van der Kloet, Louise Johns, Iona Alexander, Guy M. Goodwin, Russell G. Foster, Katharina Wulff

**Affiliations:** 1 Sleep and Circadian Neuroscience Institute, Nuffield Department of Clinical Neurosciences, University of Oxford, Oxford, UK; 2 Department of Clinical, Educational and Health Psychology, University College London, London, UK; 3 Department of Physiology, Pharmacology and Neuroscience, University of Bristol, Biomedical Sciences Building, University Walk, Bristol, UK; 4 Department of Statistics, University of Oxford, South Parks Road, Oxford, UK; 5 Norwegian Centre for Violence & Traumatic Stress Studies, University of Oslo, Oslo, Norway; 6 Department of Clinical Psychological Science, Maastricht University, Maastricht University, Maastricht, The Netherlands; 7 Department of Psychiatry, University of Oxford, Warneford Hospital, Oxford, UK; 8 Oxford Health National Health Service (NHS) Foundation Trust, Oxford, UK; 9 Departments of Radiation Sciences and Molecular Biology, Umeå University, Umeå, Sweden; 10 Wallenberg Centre for Molecular Medicine (WCMM), Umeå University, Umeå, Sweden

**Keywords:** Schizophrenia, Bipolar disorder, Human behaviour

## Abstract

Psychotic experiences (PE) are associated with poorer functioning, higher distress and the onset of serious mental illness. Environmental exposures (e.g. childhood abuse) are associated with the development of PE. However, which specific exposures convey risk for each type or dimension of PE has rarely been explored. The Oxford Wellbeing Life and Sleep (OWLS) survey includes 22 environmental risk factors for psychosis and was designed to examine how environmental risks are associated with specific dimensions of PE. Multivariate logistic regression models were fit using these risk factors to predict six dimensions of PE (perceptual abnormalities, persecutory ideation, bizarre ideas, cognitive disorganisation, delusional mood and negative symptoms). Models were built using only 70% of the data, and then fit to the remaining data to assess their generalisability and quality. 1789 (27.2% men; mean age = 27.6; SD = 10.9) survey responses were analysed. The risk factors predictive of the most PE were anxiety, social withdrawal during childhood and trauma. Cannabis and depression predicted three dimensions with both predicting bizarre ideas and persecutory ideation. Psychological abuse and sleep quality each predicted two dimensions (persecutory ideation and delusional mood). Risk factors predicting one PE dimension were age (predicting cognitive disorganisation), physical abuse (bizarre ideas), bullying and gender (persecutory ideation); and circadian phase (delusional mood). These results lend support for a continuum of psychosis, suggesting environmental risks for psychotic disorders also increase the risk of assorted dimensions of PE. Furthermore, it advocates the use of dimensional approaches when examining environmental exposures for PE given that environmental risks distribute differently across dimensions.

## Introduction

Psychotic experiences (PE) are symptoms deemed comparable to the positive symptoms of psychosis, but not sufficiently severe to warrant a diagnosis upon clinical presentation. The predominant viewpoint is that there is a phenotypic continuum of PE whereby psychosis and bipolar disorder are the most extreme manifestations along the spectrum^1^. PE are prevalent (5.8–7%), signify a susceptibility to a broad spectrum of adverse mental health outcomes and are thought to represent a transdiagnostic indicator of psychopathological severity^2–4^. As such, there is increasing recognition that PE merit their own platform for research to further understand their emergence, distribution and possible prognostic relevance (outside of their risk for the development of psychosis).

Several studies have shown that, consistent with diagnosed psychosis and schizophrenia, PE have a multidimensional structure^5,6^. The number and structure of the dimensions varies across studies (subject to analysis and measures)^6^. Genetic heritability has been shown to range from 33 to 57% depending on the dimension of PE. Allelic variants also differ in the type of PE they confer the greatest risk for ^6,7^. Dimensions also vary in their severity, associated distress and risk for the development of further mental health difficulties^6^.

### The current study

In summary, the literature tells us that not all types of PE are created equal. However, it remains poorly understood to date how assorted environmental risks (e.g. cannabis, brain injury or trauma) confer risk for different dimensions of PE. While several studies acknowledge individual environmental risk factors for the development of PE, rarely do they attempt to accumulate them to explore the environmental risk profile for a specific dimension of PE^8,9^. To help address this, we designed the Oxford Wellbeing Life and Sleep Survey (OWLS) survey to examine the prevalence of established environmental risk factors for psychosis and their efficacy in predicting the occurrence of six PE dimensions (negative symptoms, perceptual abnormalities, bizarre ideas (BI), delusional mood (DM), persecutory ideation, and cognitive disorganisation (CD)). To ensure only reliable risk factors were included, we conducted a systematic review of the meta-analyses and systematic reviews of these risk factors (Supplementary Table 1). In addition to the factors revealed by the literature review, sleep and circadian rhythm disruption has been identified as a “nonspecific” but important risk factor for psychosis, meriting its inclusion^10,11^. Thus, the goals for the present study are (1) to explore the prevalence for established risk factors for psychosis, (2) to see how these risk factors distribute across the number of PE a respondent endorses and (3) to examine how environmental risk factors distribute in their ability to predict different dimensions of PE^12^.

## Methods

### Survey sampling

The survey was targeted at the general population (aged 18–65 years) in the Oxford area. As such, advertisements were placed in a diverse number of venues and locations in Oxford, as well as online. However, as this study took place in a university city the sample was inevitably biased towards a younger cohort. Given psychosis with late-life onset has been shown to have a different risk profile to psychosis developed earlier in life and this study is designed to explore the risk factors for emerging PE (which are often during adolescence to early adulthood), a skewed age distribution was not considered a limitation in this study in the same way it may be in other survey-based studies^12^. The survey was hosted online on the Oxford University network. The survey protocol and contents were approved by the Medical Sciences Interdivisional Research Ethics Committee (MSD-IDREC-C1–2014–054), and all participants gave informed consent online when agreeing to complete the survey.

### Environmental risk factor selection

The selection of risk factors presented in the survey was premised upon a systematic ‘meta’ review of the literature, i.e. a review of the available meta-analyses and systematic reviews that globally account for the published data on a specified risk factor for the development of psychosis. Full details of the methodology employed and the risk factors identified in this process can be found in Supplementary Materials 1. Parental communication was the only risk factor highlighted by this review that could not be included, as it is evaluated by means of video recordings and there was no feasible way to replicate this accurately within the survey.

### Survey structure and instruments

#### Sociodemographic characteristics

Data were gathered on gender, age, ethnicity, education level, psychiatric disorder diagnoses, and help-seeking behaviour for any psychiatric disorder listed. An overview of each of the risk factors, sociodemographic characteristics and questionnaires evaluated in the OWLS survey is presented in Supplementary Tables 2 and 3.

#### Psychotic-like experiences

*Prodromal Questionnaire 16 Item Version* (PQ16; *α* = 0.79 for this sample)^13^. The PQ16 contains 16 items with yes/no responses, yielding a score out of 16. Scoring 6 or above warrants further screening for an at-risk mental state. The questionnaire assesses positive symptoms (perceptual abnormalities, BI, DM, paranoia and CD) and negative symptoms (social anxiety and avolition). An overview of the items and their respective dimensions can be found in Supplementary Table 4. The PQ16 was selected as it does not have ‘hypothetical qualifiers’ or describe beliefs of cultural subgroups (e.g. voodoo) which have been highlighted to produce misleading results in the estimated prevalence of PE^12,13^.

#### Axis I symptomatology

*Depression Anxiety and Stress Scale* (21 item version; *α* = 0.93 (overall); *α* = 0.93 (D); *α* = 0.80 (A); *α* = 0.87 (S)). This scale was selected as it takes a dimensional view of depression, anxiety and stress. It can be subdivided into three categories (of seven items each). The 21 items are each scored on a 4-point scale from 0 to 3^14^.

#### Traumatic events

*Life-Threatening Experiences Scale* (LTE)^15^. The LTE is a 12-item self-report questionnaire assessing different categories of traumatic life events, selected for their established long-term consequences^15^. The total score is the number of items endorsed (maximum = 12). Participants are asked to rate their level of stress associated with each event endorsed, choosing one of four options (not stressful, slightly stressful, moderately stressful, and very stressful).

#### Sleep quality

*Pittsburgh Sleep Quality Index* (PSQI; *α* = 0.82)^16^. The PSQI measures subjective sleep quality over the previous month, yielding a score ranging from 0 to 21. Higher scores represent poorer quality sleep^17^.

#### Insomnia

*Short Form Sleep Condition Indicator* (SCI; *α* = 0.82)^18^. The SCI consists of two items: (1) ‘thinking about a typical night in the last month, how many nights a week do you have a problem with your sleep?’; and (2) ‘thinking about the past month, to what extent has poor sleep troubled you in general?’. Possible responses to the first question are ‘0–1’, ‘2’, ‘3’, ‘4’ and ‘5–7’; and to the second question are ‘not at all’, ‘a little’, ‘somewhat’, ‘much’ and ‘very much’. Both answer sets are scored 4, 3, 2, 1 and 0, respectively. The two scores are added to give the SCI score. Lower scores indicate more aggressive insomnia complaints^18^.

#### Circadian phase

*The Munich Chronotype Questionnaire* (MCTQ)^19^. The MCTQ assesses habitual sleep-wake timing from bedtime to wake-up time and is based on subjects’ judgement of their sleep habits over the last 2 weeks. These timings are assessed separately for work and free days. The responses produce a time-based variable, the mid-sleep point on free days (MSF), which is then corrected for accumulated sleep deficits (MSFsc) during the work-week. The corrected variable, MSFsc, provides a correlate of circadian phase in local clock time with a later mid-sleep point indicating a later circadian phase (synonymous with late or ‘evening chronotype’).

#### Other environmental risk factors

Based upon the results of the systematic review, the following risk factors were included: infections of the brain; brain injury; cannabis use; childhood abuse (questions taken from Cuijpers et al. 2011)^20^; childhood bullying; childhood social withdrawal (social withdrawal subscale items 42, 65, 88 and 111 from the Child Behavioural Checklist edited to make appropriate for retrospective report); family history of psychiatric disorders; migrant status; help-seeking behaviours in relation to the PE; latitude position at birth; diagnosis of epilepsy; obstetric complications at birth; paternal age; season of birth; presence of 22q11.2 deletion syndrome diagnosis; and urbanicity (Supplementary Tables 2 and 3)^21,22^.

### Statistical analyses

Before the analyses, all survey data were subjected to quality control. A detailed breakdown of the cleaning process is provided in Supplementary Materials 2. All subsequent statistical analyses were performed within the R statistical environment (Version 3.4.1). Cross-sectional differences were explored across four predefined PE risk levels according to the number of PE endorsed in the sample: (1) a PQ16 score of 0, indicating a very low risk; (2) a PQI6 score between PE 1 and 5, indicating minimal risk; (3) a PQ16 score above 5 (the established cut-off for the PQ16), indicating a moderate or ‘at-risk’ group; and (4) a PQ16 score above 5 and having sought help and with distress associated with these symptoms, indicating the highest risk group. These groups were designed to examine the continuity of risk for PE across the respondents.

To examine specific risk factors for the six dimensions of PE, we built multivariate logistic regression models, using the set of risk factors, demographics, sleep variables, and psychopathology measures as predictor variables. The ‘negative symptoms’ PQ16 dimension was modelled first as a proof of concept, as many of the predictor variables relate to the presence of a mood disorder or depressive/anxious symptomatology. As this model indeed had high predictive power, we extended this approach to the other dimensions.

Given the large number of possible combinations of the predictor variables for inclusion in each model, we performed automated model selection using the Akaike information criterion (AIC) to objectively provide a set of candidate models for further consideration. The AIC measures the relative quality of a collection of models and penalises model complexity, thus discouraging overfitting.

For model quality assessment, and to further discourage overfitting, we built models using 70% of the responses. These ‘training’ data were randomly selected. Upon finalisation, the models were then fit to the remaining 30% (the ‘test’ data), to provide an assessment of their generalisability based on the quality of model fit to data the models had not seen. To measure model accuracy, we calculated classification success rates with both training and test data (i.e. the models’ ability to predict the known response data), and computed receiver operating characteristic (ROC) curves. A more detailed overview of the modelling process and the model quality evaluation can be found in Supplementary Materials 3.

## Results

### Demographics and distribution of risk factors partitioned by the number of PE endorsed

The sample (*n* = 1789, 487 men) had a mean age of 27.6 years (range: 16–65). Just under half (*n* = 789, 44.1%) had a low to medium level of education (completed secondary school at most), while over half the sample (*n* = 1000; 55.9%) possessed an undergraduate degree or a postgraduate higher qualification (MA or PhD). Many were studying towards a higher level of education (*n* = 1234; 69.0%), indicating this to be a highly educated sample. A demographic overview partitioned by number of PE endorsed is provided in Table 1.

**Table 1:** Demographic overview of survey respondents according to the number of psychotic symptoms endorsed (*n* = 1789).

	None	1–5	At risk	High risk	Overall
*n*	372	1053	220	144	1789
Age (SD)	29.9 (11.6)	27.7 (11)	24.8 (9.2)	24.9 (9)	27.6 (10.9)
Males (%)	115 (30.9)	293 (27.8)	57 (25.9)	22 (15.3)	487 (27.2)
Level of education					
Low	2 (0.5)	20 (1.9)	19 (8.6)	7 (4.9)	48 (2.7)
Med	106 (28.5)	437 (41.5)	121 (55)	77 (53.5)	741 (41.4)
High	126 (33.9)	281 (26.7)	39 (17.7)	27 (18.8)	473 (26.4)
Very high	138 (37.1)	315 (29.9)	41 (18.6)	33 (22.9)	527 (29.5)
Studying	244 (65.6)	721 (68.5)	167 (75.9)	102 (70.8)	1234 (69)
Studying BA/BSc	95 (25.5)	379 (36)	96 (43.6)	58 (40.3)	628 (35.1)
Diagnoses^a^					
Depression	22 (5.9)	191 (18.1)	50 (22.7)	85 (59)	348 (19.5)
MDD	3 (0.8)	22 (2.1)	4 (1.8)	14 (9.7)	43 (2.4)
Social anxiety	1 (0.3)	19 (1.8)	12 (5.5)	20 (13.9)	52 (2.9)
Health anxiety	1 (0.3)	6 (0.6)	0 (0)	3 (2.1)	10 (0.6)
OCD	0 (0)	18 (1.7)	3 (1.4)	8 (5.6)	29 (1.6)
BDD	0 (0)	9 (0.9)	8 (3.6)	5 (3.5)	22 (1.2)
GAD	7 (1.9)	62 (5.9)	16 (7.3)	35 (24.3)	120 (6.7)
Panic	3 (0.8)	13 (1.2)	5 (2.3)	9 (6.2)	30 (1.7)
Alcohol/sub.	1 (0.3)	1 (0.1)	0 (0)	3 (2.1)	5 (0.3)
Phobia	0 (0)	2 (0.2)	2 (0.9)	4 (2.8)	8 (0.4)
Other	5 (1.3)	51 (4.8)	19 (8.6)	30 (20.8)	105 (5.9)
Treatment					
Counselling^a^	28 (7.5)	206 (19.6)	55 (25)	94 (65.3)	383 (21.4)
Medication^a^	23 (6.2)	187 (17.8)	46 (20.9)	83 (57.6)	339 (18.9)
Hospitalisation^a^	1 (0.3)	20 (1.9)	7 (3.2)	17 (11.8)	45 (2.5)
Untreated	2 (0.5)	12 (1.1)	3 (1.4)	4 (2.8)	21 (1.2)
Psychometric profile					
Psychotic exp^b^	0	2	7	7	2
PE—distress^b^	0	2	8	12	2
Depression^b^	4	10	18	26	10
Anxiety^b^	2	6	12	18	6
Stress^b^	8	12	20	26	14
Help seeking	0 (0)	197 (18.7)	0 (0)	144 (100)	341 (19.1)
Ethnicity					
White	311 (83.6)	910 (86.4)	178 (80.9)	130 (90.3)	1529 (85.5)
Asian	36 (9.7)	72 (6.8)	23 (10.5)	5 (3.5)	136 (7.6)
Arabic	2 (0.5)	2 (0.2)	1 (0.5)	3 (2.1)	8 (0.4)
Black	5 (1.3)	5 (0.5)	1 (0.5)	1 (0.7)	12 (0.7)
Mixed	13 (3.5)	46 (4.4)	15 (6.8)	4 (2.8)	78 (4.4)
Other	5 (1.3)	18 (1.7)	2 (0.9)	1 (0.7)	26 (1.5)

Cross-sectional differences were explored across four predefined PE risk levels according to the number of psychotic experiences endorsed in the sample: (1) a PQ16 score of 0, indicating a very low risk; (2) a PQI6 score between 1 and 5, indicating minimal risk; (3) a PQ16 score above 5 (the established cut-off for the PQ16), indicating a moderate or ‘at-risk’ group; and (5) a PQ16 score above 5 and having sought help or experienced distress associated with these symptoms, indicating the highest risk group. Low to medium level of education refers to completing secondary school. High is possessing an undergraduate and very high is possessing a postgraduate qualification. Studying refers to participants currently studying towards a higher level of education. Diagnoses, treatments and ethnicities are reported as percentages of the sample whereas the psychometric profile (with the exception of help seeking) uses the mean or median score across a group.

^a^Showed significant differences between risk levels (see Supplementary Table 5 for more details).

^b^Presented with a skewed distribution, as such, the median was used as a measure of central tendency. Treatment respondents can put down more than one response.

**Fig. 1:**
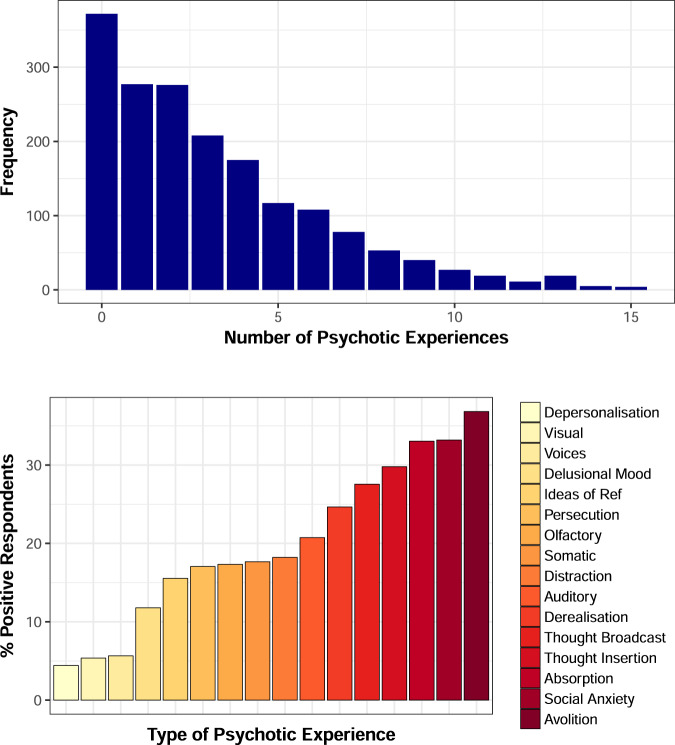
Histogram highlighting the prevalence and types of PE endorsed in this sample. The histogram in blue highlights that at least one PE was endorsed by the majority of the sample (79.2%). The histogram has a sharp downward curve as the number of PEs endorsed rises with 20.3% endorsing 5 or more PEs. The histogram in red highlights which types of PE saw the highest rates of endorsement. The most endorsed PE was Avolition (akin to depression; 36.8%), which was closely followed by social anxiety (33.2%), absorption (33.0%), thought insertion (29.8%) and thought broadcasting (27.6%). This supports the notion of a continuum of PE seen in the normal population.

At least one PE was endorsed by 1417 respondents (79.2% of sample). Of these, 364 (20.3%) endorsed a PQ16 above 5. Overall, 144 (8.1%) respondents endorsed a PQ16 above 5 with associated distress and help-seeking behaviour specific to the PE (Fig. 1; top). The most common experience endorsed was avolition (akin to depression; 36.8%), which was closely followed by social anxiety (33.2%), absorption (33.0%), thought insertion (29.8%) and thought broadcasting (27.6%; Fig. 1; bottom).

**Fig. 2:**
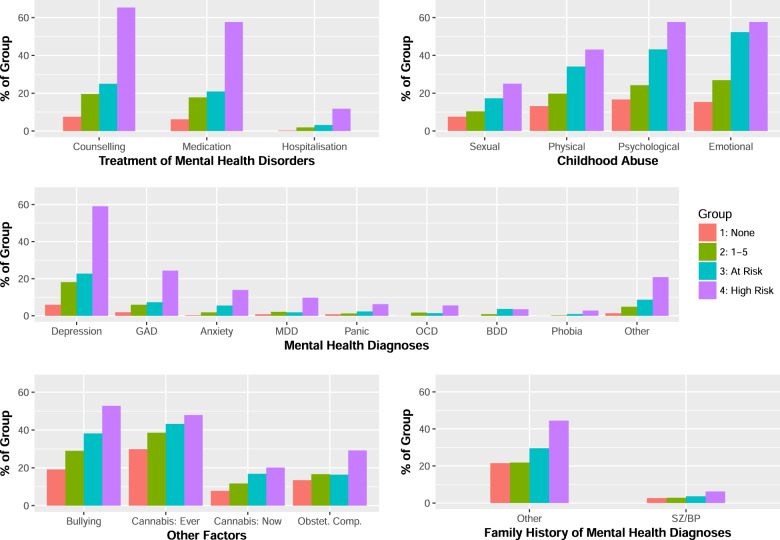
Bar charts highlighting the prevalence of risk factors with respect to the number of PE endorsed. An increasing prevalence across the four predefined PE risk groups was observed for all risk factors for which a sufficient incidence rate was present. Among these 22 variables, chi-square tests revealed that all risk factors were unequally distributed between the risk groups. Demographic factors (ex: ethnicity), however, were not significantly differently distributed across the risk groups.

An increasing prevalence across the four predefined PE risk groups was observed for all risk factors for which a sufficient incidence rate was present (Fig. 2; Tables 1, 2). Among these 22 variables, chi-square tests revealed that all risk factors were unequally distributed between the risk groups including adverse childhood experiences, cannabis use, the need for care, and diagnoses of non-psychotic mental health disorders. Demographic factors, however, were not significantly differently distributed across the risk groups (Supplementary Table 5). Altogether, these data imply that OWLS survey respondents replicate observations based on the psychosis-proneness continuum concept.

**Table 2:** Distribution of risk factors across different risk levels (*n* = 1789).

	None	1–5	At risk	High risk	Overall
*n*	372	1053	220	144	1789
Age (SD)	29.9 (11.6)	27.7 (11)	24.8 (9.2)	24.9 (9)	27.6 (10.9)
Males (%)	115 (30.9)	293 (27.8)	57 (25.9)	22 (15.3)	487 (27.2)
Genetic and developmental risks					
FH (SZ/BP)^a^	10 (2.7)	30 (2.8)	8 (3.6)	9 (6.2)	57 (3.2)
FH (Other)^a^	80 (21.5)	230 (21.8)	65 (29.5)	64 (44.4)	439 (24.5)
Paternal age	45 (12.1)	120 (11.4)	39 (17.7)	16 (11.1)	220 (12.3)
Obstetric C^a^	50 (13.4)	175 (16.6)	36 (16.4)	42 (29.2)	303 (16.9)
Season of birth	185 (49.7)	532 (50.5)	103 (46.8)	74 (51.4)	894 (50)
Latitude (North)	13 (3.5)	25 (2.4)	4 (1.8)	3 (2.1)	45 (2.5)
22Q11.2	0 (0)	0 (0)	0 (0)	0 (0)	0 (0)
Migrant history	96 (25.8)	229 (21.7)	44 (20)	30 (20.8)	399 (22.3)
Migrant 1st G	5 (1.3)	13 (1.2)	2 (0.9)	0 (0)	20 (1.1)
Migrant 2nd G	7 (1.9)	40 (3.8)	6 (2.7)	5 (3.5)	58 (3.2)
Sleep risks					
PSQI^b^	4	5	7	8	5
PSQI > 5^a^	85 (22.8)	508 (48.2)	158 (71.8)	121 (84)	872 (48.7)
SCI^b^	7	6	4	2	5
SCI < 4^a^	67 (18)	288 (27.4)	96 (43.6)	96 (66.7)	547 (30.6)
Circadian phase	4.08	4.36	4.66	4.5	4.34
Childhood risks					
Bullying^a^	71 (19.1)	305 (29)	84 (38.2)	76 (52.8)	536 (30)
Social W score	1	2	4	4	2
Social W^a^	268 (72)	882 (83.8)	206 (93.6)	137 (95.1)	1493 (83.5)
Physical abuse^a^	49 (13.2)	208 (19.8)	75 (34.1)	62 (43.1)	394 (22)
Sexual abuse^a^	28 (7.5)	109 (10.4)	38 (17.3)	36 (25)	211 (11.8)
Psych. abuse^a^	62 (16.7)	255 (24.2)	95 (43.2)	83 (57.6)	495 (27.7)
Emot. abuse^a^	57 (15.3)	283 (26.9)	115 (52.3)	83 (57.6)	538 (30.1)
Trauma					
Prop^N^ trauma^a^	324 (87.1)	982 (93.3)	218 (99.1)	141 (97.9)	1665 (93.1)
Trauma event	3	3	4	4	3
Trauma distress	2	2	3	3	2
Adolescent/adult risk					
Urbanicity	100 (26.9)	264 (25.1)	52 (23.6)	27 (18.8)	443 (24.8)
Brain injury	6 (1.6)	17 (1.6)	3 (1.4)	3 (2.1)	29 (1.6)
Brain infection	3 (0.8)	4 (0.4)	1 (0.5)	1 (0.7)	9 (0.5)
Cannabis ever^a^	111 (29.8)	406 (38.6)	95 (43.2)	69 (47.9)	681 (38.1)
Cannabis now^a^	29 (7.8)	123 (11.7)	37 (16.8)	29 (20.1)	218 (12.2)
Epilepsy	0 (0)	10 (0.9)	4 (1.8)	3 (2.1)	17 (1)

FH (SZ/BP) = family history of schizophrenia or bipolar disorder; FH (other) = family history of any other serious mental illness. Family history was counted using first-degree relatives only, and cannabis (now) refers to participants endorsing using cannabis in the past 3 months at a frequency of once a month or more. The risk factor season of birth is when a participant was born in winter or spring. Genetic and developmental risks, childhood risks and adolescent/adult risks are reported as percentages of the sample (unless otherwise indicated), whereas sleep risks and trauma uses the mean or median score across a group.

*Social W* social withdrawal, *PSQI* Pittsburgh Sleep Quality Index, *SCI* Sleep Condition Indicator.

^a^Showed significant differences between risk levels (see Supplementary Table 5 for more details).

^b^Presented with a skewed distribution, as such, the median was used as a measure of central tendency.

### Examining model performance for the PQ16 dimensions

**Fig. 3:**
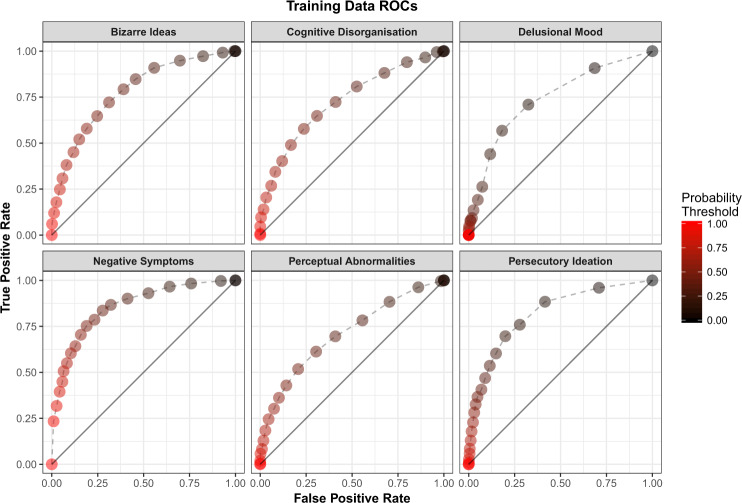
Receiver operating characteristic (ROC) curves for each of the six logistic regression models for survey respondents in the training dataset. For each plot, the points show how the true positive rate (sensitivity) and the false positive rate (1-specificity) vary as the threshold for classification into the two groups is varied.

Multivariate logistic regression models were built for the six PE dimensions (Supplementary Materials 3). Correct classification rates ranged between 66 and 79% across the six models for both the training and test data (Supplementary Table 6). ROC curves (and their AUC values) showed that each model reliably discriminated between participants who endorse and those who do not endorse at least one item for that PE dimension (Fig. 3, Supplementary Table 6).

### The relationship between risk factors and PE dimensions

**Fig. 4:**
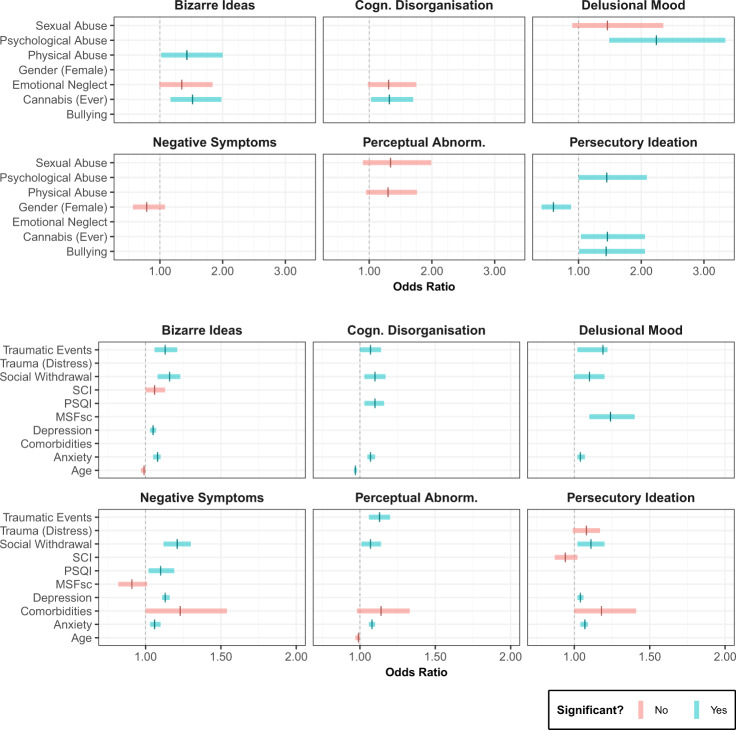
Odds ratios and corresponding confidence intervals for the modelled risk factors for the different dimensions of PE. For each model, the risk factors that significantly increase or decrease the probability of endorsing psychotic symptoms are displayed with green confidence intervals. Risk factors that do not significantly impact this probability (but are included in the model of best fit) are displayed with red confidence intervals. If the risk factor is not included in the selected model, no odds ratio is presented. The top six windows display the binary risk factors (e.g. using cannabis), which possess much wider CIs than the continuous risk factors (e.g. depression scores) displayed in the bottom six windows.

The risk factors included in each of the selected PE dimension models are detailed in Table 3. The odds ratios associated with each risk factor in the models are represented in Fig. 4.

**Table 3:** Model summaries for negative symptoms, perceptual abnormalities, persecutory ideation, bizarre ideas, delusional mood and cognitive disorganisation.

	OR (95% CI)	Estimate	SE	Z	*p*
Negative symptoms					
Intercept	−	−2.15	0.32	−6.73	<0.0001
Anxiety	1.06 (1.03–1.10)	0.06	0.01	3.88	0.0001
Social withdrawal	1.21 (1.12–1.30)	0.19	0.04	4.90	<0.0001
Depression	1.13 (1.11–1.16)	0.12	0.01	10.22	<0.0001
Sleep quality (PSQI)	1.10 (1.02–1.19)	0.09	0.04	2.40	0.0165
Circadian phase (MSFsc)	0.91 (0.82–1.01)	−0.09	0.05	−1.725	0.0845
Gender (Female)	0.79 (0.57–1.08)	−0.24	0.16	−1.48	0.1389
Comorbidities	1.23 (1.00–1.54)	0.21	0.11	1.90	0.0580
Perceptual abnormalities					
Intercept	–	−1.46	0.22	−6.68	<0.0001
Anxiety	1.08 (1.06–1.10)	0.08	0.01	8.18	<0.0001
Traumatic events	1.13 (1.06–1.20)	0.12	0.03	3.70	0.0002
Social withdrawal	1.07 (1.01–1.14)	0.07	0.03	2.17	0.0302
Physical abuse	1.30 (0.95–1.76)	0.26	0.16	1.66	0.0963
Comorbidities	1.14 (0.98–1.33)	0.13	0.08	−1.64	0.1013
Age	0.99 (0.97–1.00)	−0.01	0.01	−1.86	0.0634
Sexual abuse	1.34 (0.90–1.99)	0.30	0.20	1.43	0.1538
Persecutory ideation					
Intercept	–	−3.23	0.36	−8.93	<0.0001
Anxiety	1.07 (1.04–1.09)	0.06	0.01	5.13	<0.0001
Social withdrawal	1.11 (1.02–1.20)	0.10	0.04	2.34	0.0194
Cannabis (ever)	1.46 (1.04–2.06)	0.38	0.17	2.17	0.0302
Depression	1.04 (1.02–1.06)	0.04	0.02	4.50	<0.0001
Psychological abuse	1.45 (1.00–2.09)	0.37	0.20	1.98	0.0479
Bullying	1.44 (1.01–2.06)	0.37	0.18	2.01	0.0448
Gender (female)	0.60 (0.41–0.88)	−0.51	0.20	−2.60	0.0094
Comorbidities	1.18 (1.00–1.41)	0.17	0.09	1.91	0.0559
Trauma (Distress)	1.08 (0.99–1.17)	0.08	0.04	1.73	0.0796
Insomnia (SCI)	0.94 (0.87–1.02)	−0.06	0.04	−1.52	0.1285
Bizarre ideas					
Intercept	–	−2.20	0.34	−6.42	<0.0001
Anxiety	1.08 (1.05–1.10)	0.073	0.01	5.88	<0.0001
Social withdrawal	1.16 (1.08–1.23)	0.145	0.04	4.151	<0.0001
Traumatic Events	1.13 (1.06–1.21)	0.124	0.03	3.64	<0.0001
Cannabis (Ever)	1.52 (1.17–1.98)	0.418	0.13	3.12	0.0018
Depression	1.05 (1.03–1.07)	0.046	0.01	5.29	<0.0001
Physical abuse	1.43 (1.02–2.00)	0.355	0.17	2.06	0.0391
Age	0.99 (0.97–1.00)	−0.013	0.01	−1.94	0.0530
Insomnia (SCI)	1.06 (1.00–1.13)	0.062	0.03	1.92	0.0554
Emotional neglect	1.35 (0.99–1.84)	0.301	0.16	1.90	0.0571
Delusional mood					
Intercept	–	−4.59	0.38	−12.20	<0.0001
Anxiety	1.04 (1.02–1.07)	0.04	0.11	3.68	0.0002
Social withdrawal	1.10 (1.00–1.20)	0.09	0.05	2.02	0.0438
Traumatic events	1.19 (1.02–1.22)	0.11	0.04	2.52	0.0118
Psychological abuse	2.24 (1.49–3.34)	0.80	0.20	3.93	<0.0001
Circadian phase (MSFsc)	1.24 (1.10–1.40)	0.22	0.06	3.60	0.0003
Sexual abuse	1.46 (0.90–2.35)	0.38	0.25	1.56	0.1190
Cognitive disorganisation					
Intercept	–	−0.98	0.24	−4.08	<0.0001
Anxiety	1.07 (1.05–1.10)	0.07	0.01	6.42	<0.0001
Social withdrawal	1.10 (1.03–1.17)	0.09	0.03	2.76	0.0058
Traumatic events	1.07 (1.00–1.14)	0.07	0.03	2.10	0.0375
Cannabis (Ever)	1.32 (1.03–1.70)	0.28	0.13	2.15	0.0314
Sleep quality (PSQI)	1.10 (1.03–1.16)	0.09	0.03	2.81	0.0050
Age	0.97 (0.96–0.98)	−0.03	0.01	−4.61	<0.0001
Emotional neglect	1.31 (0.98–1.75)	0.27	0.15	1.84	0.0658

The risk factors are ordered in the number of PE dimensions they share (first) and in alphabetical order (second). Comorbidities refer to the endorsement of any mental health diagnosis excluding those of psychotic disorders.

For negative symptoms (Table 3), depression, anxiety, social withdrawal and sleep quality were found to be significant predictor variables. The odds ratios imply a one-point increase in depression, anxiety, social withdrawal or poorer sleep quality was associated with a respective 13%, 6%, 21% and 10% increase in the probability of endorsing negative symptoms, assuming all other variables are kept constant (Fig. 4).

Perceptual abnormalities were best predicted by anxiety, traumatic events and social withdrawal. A one-point increase in anxiety, number of traumatic events endorsed or social withdrawal was associated with a respective 8%, 13% and 7% increases in the probability of endorsing perceptual abnormalities.

Persecutory ideation was predicted by depression, anxiety and social withdrawal, with one-point increases associated with respective 4%, 7% and 11% increases in the probability of endorsing persecutory ideation. Furthermore, psychological abuse, having used cannabis and bullying were all found to significantly increase the probability of endorsing persecutory ideation by 45%, 46% and 44%, respectively, assuming all other variables are kept constant. Participants identifying as female had a reduced risk (OR = 0.60, 0.41–0.88, *p* = 0.0094), being 40% less likely to endorse persecutory ideation than men. Comorbidities and distress from traumatic events did not significantly impact the probability of endorsing persecutory ideation (*p* = 0.06 and 0.08, respectively).

Like negative symptoms and persecutory ideation, BI were associated with by depression, anxiety, traumatic events and social withdrawal, with 5, 8, 13 and 16% increases in the probability of endorsing BI for a one-point increment in each of the respective predictors. Furthermore, physical abuse during childhood and cannabis use were found to be significant predictors, with a respective 52% and 43% increased probability of endorsing BI for those who have used cannabis or experienced physical abuse. Small effects of insomnia (SCI) and emotional neglect were also observed.

DM was predicted by anxiety, social withdrawal and traumatic events, with one-unit increases in the predictor variables leading to 4%, 10% and 19% respective increases in the probability of endorsing DM. Other significant predictors for DM were psychological abuse and circadian phase (MSFsc).

Finally, CD was predicted by anxiety, social withdrawal and traumatic events. Having used cannabis also predicted CD, with cannabis users having an estimated 32% increased probability of endorsing CD. Furthermore, CD was also predicted by sleep quality and age, with a one-point decrease in sleep quality predicting a 10% increased probability of endorsing CD, and with a 1-year age increase predicted to give a 3% decrease in the probability of endorsing CD.

**Fig. 5:**
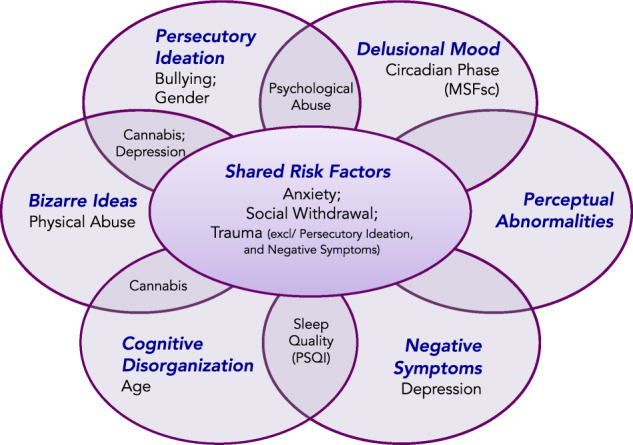
Six psychotic experience dimensions with predictive overlapping and distinct risk factors.

The overall contribution and overlap of factors predicting each PE dimension is presented in Fig. 5. Ethnicity, season of birth, paternal age, urbanicity, first-degree relatives with a family history of mental illness, presence of any psychiatric comorbidity or the treatment for comorbid mental health problems did not significantly predict an increased (or decreased) risk of any of the PE dimensions (Figs. 4 and 5).

## Discussion

This cross-sectional survey examined how risk factors for psychosis distribute across the number of PE a respondent endorses and whether heightened PE indicates heightened need for care. It then sought to further explore whether environmental risk factors predict different dimensions of PE.

Our results support the hypothesis that PE present as heterogeneous, subclinical features in the general population, with certain experiences more abundantly endorsed (i.e. avolition and social anxiety) than others (i.e. visual, auditory or olfactory sensory-perceptual abnormalities). The results also lend further support for the phenotypic continuum of PE with those reporting higher numbers of PE also reporting more mental health diagnoses, sleep disturbances and need for care (Table 1). Furthermore, childhood risks (e.g. sexual abuse and bullying), a family history, obstetric complications, cannabis use and exposure to trauma also appear to be more prevalent in those who endorse higher numbers of PE (Table 2; Supplementary Table 5), which again would support the notion of a continuum and indicate that endorsing more PE might indicate heightened psychopathological severity. As this is cross-sectional data, this must be interpreted with caution and causal inferences are not possible.

While many environmental risk factors have been described to increase the likelihood of developing psychotic and affective symptoms upon their exposure and over time^23^, to date there have been no reports of environmental risk factors differentially predicting assorted dimensions of PE in adults. We sought to bring clarity to this by examining probability models and their reliability in correctly identifying the occurrence of dimensionally assessed PE. Figure 5 illustrates the distribution of risk factors across the six dimensions of PE.

Overall, we found that anxiety and social withdrawal during childhood are shared across all six dimensions. Traumatic life events predicted the occurrence of four dimensions (BI, DM, CD and perceptual abnormalities). These results are perhaps unsurprising given all three risk factors have long been hailed as important triggers for psychosis^12^. Furthermore, both childhood trauma and stressful life events appear to contribute to the persistence of PE over time^24^.

Depressive symptoms and cannabis use each predicted three dimensions of PE. Importantly, cannabis was not found to predict perceptual abnormalities or DM, commonly regarded as the most discernible symptoms of psychosis. One explanation for this may be the low numbers of perceptual abnormalities endorsed, or perhaps that cannabis presents as a risk factor for only certain dimensions of PE.

Risk factors that uniquely predicted the occurrence of a single PE dimension were childhood physical abuse for bizarre ideation, bullying for persecutory ideation and circadian phase for DM. Protective risk factors were age (decreasing the risk of CD) and gender (with women at a lower risk of persecutory ideation). Overall, childhood abuse (psychological and physical) and cannabis presented the highest severity of risk.

Sleep phenotypes distributed differently across different dimensions. Poor sleep quality increased the probability of endorsing negative symptoms and CD, yet late circadian phase predicted the occurrence of DM. That sleep quality and circadian phase are predictive of different PE dimensions is not unexpected given the heterogeneous sleep-wake phenotypes observed in patients diagnosed with schizophrenia and bipolar disorder^25,26^. This may relate to the variants of circadian clock genes which produce different physiological susceptibilities and phenotypic effects, independent from sleep regulation. Given this survey is cross-sectional and only contains subjective report, considerably more research is warranted to substantiate this claim.

CD in our survey is similar to the core characteristics of dissociative experiences, which appear to show strong ties to sleep^27^. Given the suggested mediating role of dissociative experiences on auditory hallucinations^28^, they present an interesting avenue to investigate the role of sleep in the development of PE^29^.

The analyses outlined here present broader implications for the differences in outcomes found between cross-sectional observations and regression analyses. On a cross-sectional basis, differences in group prevalence (number of psychotic symptoms and help seeking) are seen for most risk factors examined here (e.g. family history, obstetric complications, child abuse, etc.), except for urbanicity, ethnicity, season of birth, migrant status and paternal age. However, only about half of the risk factors translated to the multivariate regression models. Psychiatric diagnoses, which presented stark differences cross-sectionally and family history, a widely accepted genetic risk, did not significantly predict the occurrence of any dimension of PE. Similar differences have been reported before. For example, Cannon et al. reported that despite being established risk factors for psychosis, stressful life events, traumas, age and family history, did not predict actual transition to a psychotic episode using their individualised risk calculator^30^. However, family history of psychosis had a very pronounced effect on the onset of schizophrenia and non-affective psychosis in another study, while urbanicity had no effect^12^. Similarly, the impact of a winter/spring birth on the prevalence of schizophrenia has been highlighted in a meta-analysis^31^, yet negative results have been reported for impact of a winter/spring birth on the detection of PE^32^.

Given the heterogeneity across studies in what predicts risk, the results presented here suggest that variation may result (in part) from a difference in high/low affinity between risk factors and dimensions of PE. More refined approaches could certainly help inform why psychosis-spectrum disorders are so heterogeneous in their clinical presentation, and why individuals develop PE subsequently to mental health disorders (and vice versa)^3^. More broadly, dimensional research (including the study described here) is integral for the implementation of personalised medicine within the treatment of mental health disorders. Schematics (such as Fig. 5) could provide helpful roadmaps to clinicians as to what risks most often co-occur with which symptoms or PE. In turn, more targeted interventions to the symptomatic profile of the patient could be offered.

### Limitations

While this study has many strengths, i.e. comprehensive risk factor search, uniform data collection and high-resolution questionnaires, it has also a number of important limitations. Students are overrepresented in this sample and we did not examine the relationship status of respondents, both of which have been shown to be associated with endorsement of PE and limit generalisability^2^. The negative symptoms dimension (which is comprised of two items examining anhedonia and social anxiety) is primarily predicted by depression and anxiety symptoms (alongside sleep quality and social withdrawal during childhood). This model was first run as a proof of concept to explore the viability of dimensional analyses but is limited in that the predictors are correlates of the outcome measure. Geographical dispersion and ethnical dispersal is low for this survey, both have also been shown to impact the prevalence of PEs^2,12^. Risk factors, such as alcohol and psychoactive drugs, as well as cognitive abilities and genetic risk factors (other than family history), all known to increase the risk of psychosis and PEs could not be examined in survey format^30^. We relied on self-report that has been shown to overestimate the occurrence of PEs, thereby not permitting cross-examination of reported experiences being indeed psychotic^33^. The questionnaires addressed whether a certain experience occurred during the last year but not the number of times it was experienced, which can vary to a considerable degree^33^. Finally, the rates of PE presented here are above what has been previously reported using other instruments (7–12%) but are in line with previous reports of the same PE questionnaire in similar populations^23,34^. This could reflect a sampling bias, as the survey was advertised as relating to wellbeing or it could be reflective of the wording of the PQ16, which encourages a more liberal response style comparative to other measures, or indeed, could reflect both^23^. As with any cross-sectional multidimensional dataset, causality cannot be inferred from the predictive direction of effects in the models described here. Furthermore, it is possible that effects of the environment may involve genes or gene-environment interactions or correlations (which again cannot be captured within the results presented here).

## Conclusions and future directions

The OWLS survey is the first of its kind: a survey designed to tackle the question of whether empirically robust environmental risk factors can predict the occurrence of dimensionally assessed PE, or whether the majority of these risks are only observable using cross-sectional comparisons. Previous studies have used larger samples but lower resolution measures, or have reanalysed national survey data, but the OWLS survey is the first to specifically target risk factors for psychosis and examine in detail what their relationship holds to PE dimensions. It is also the first survey to examine the independent roles sleep and circadian phase may play in the dimensionally assessed psychotic symptoms using high-resolution measures and modelling analyses, which deserves greater attention. Further research aimed at replicating the specificity of risk factors to certain dimensions of PE may be of real benefit to understanding the heterogeneity of presentations observed in clinical practice. Future work should consider longitudinal follow-up surveys to understand the role of risk factors in predicting outcomes, including transition to psychosis, other mental health diagnoses, number of PEs, and need for care.

## Supplementary information

Supplementary Materials
