# Supplementary material for: Do environmental risk factors for the development of psychosis distribute differently across dimensionally assessed psychotic experiences?
Source: Transl Psychiatry. 2021 Apr 19;11:226. doi: 10.1038/s41398-021-01265-2 (PMC8055691; doi:10.1038/s41398-021-01265-2)
Supplement: Supplementary file 1 — Supplementary Materials [file 41398_2021_1265_MOESM1_ESM.pdf]

Supplementary Materials 1 - Literature Review and Methodology

Systematic Review of the Risk Factors for Psychosis
A systematic search was conducted to identify meta-analyses and systematic reviews reporting on associations between risk factors and psychosis.

1.1 Search Strategy

MEDLINE, PsycINFO, Embase, and Global Health databases were searched from inception to February 2014. Both title/abstract mapping (denoted by ti,ab.) and thesaurus mapping (articles selected based upon database-specific subject headings) were used. To identify articles looking into risk factors for psychosis, the following search terms were used: psychosis*.ti,ab. OR psychotic*.ti,ab. OR schizo*.ti,ab. AND risk*.ti,ab. OR suscept*.ti,ab. OR premorbid*.ti,ab. OR predispos*.ti,ab. OR vulnerab*.ti,ab. OR antecedent*.ti,ab. OR precursor*.ti,ab. (title/abstract mapping); exp risk factors/, or exp at risk populations/, or exp predisposition/, exp "susceptibility (disorders)"/, or exp premorbidity/ (thesaurus mapping). Finally, the risk factor search terms were limited to systematic reviews or meta-analyses ('risk factor search terms' AND 'psychosis search terms' AND 'systematic review*.ti,ab. OR meta analys*.ti,ab.'). Supplementary database searching was also used to identify additional articles on specific risk factors for psychosis.

1.2 Inclusion/Exclusion Criteria

We conducted the search with progressive increases in inclusion/exclusion criteria for each stage of screening. This included a title screen, abstract screen, and full article screen. Disagreements on including or excluding articles were resolved through group discussion.

Articles were searched for regardless of their publication status or language. To be eligible for inclusion, articles had to report that they were a systematic review or meta-analysis in the title, and report pooled data (e.g. OR, RR, effect size, or prevalence rates) for the factor of interest. Factors of interest were considered 'risk factors' and subsequently included in review if they met the a priori definition of being any attribute, characteristic, or exposure which increases the likelihood of developing the disorder, but not being associated with that disorder's symptomatology or associated as a comorbid condition. Thus, traits such as cognitive impairment were not included in our main analyses. Since this study is focused on environmental influences on psychosis, articles were excluded if they reported genetic risk factors for specific genes, risk factors at the level of neuronal function, brain activity or brain morphology, and animal based models.

1.3 Result

After a full article screen, 30 articles (27 meta-analyses and 3 systematic reviews) were included (see the flowchart on the following page). From these papers, we identified 20 risk factors which were: presence of a family history of psychiatric disease, ethnicity, obstetric complications, parenting, child abuse (physical, emotional and sexual), migration status, cannabis use, traumatic life events, bullying, brain injury, Axis I diagnosis, gender, latitude and climate, paternal age, prenatal infections, season of birth, social withdrawal, urbanicity, childhood viral infections and

22q.1 deletion syndrome. An overview of these papers can be found in **Supplementary Table 1**.

A further two references investigating cannabis use are not included in the aforementioned table as one investigated the potential of an advance in the age of onset for psychosis (Large, Sharma, Compton, Slade, & Nielssen, 2011) and the other investigated the comorbidity of cannabis use disorders in schizophrenia (Koskinen, Löhönen, Koponen, Isohanni, & Miettunen, 2009). As these studies did not set out to directly investigate the impact of smoking cannabis on the risks of developing a psychotic disorder, they were excluded (while cannabis use itself is considered a risk factor). A further paper on obstetric complications by Geddes et al., (1995) was also not listed as it is substantially older than that of Cannon et al., (2002). For a detailed breakdown on how each of the risk factors are measured, refer to **Supplementary Tables 2 & 3**.

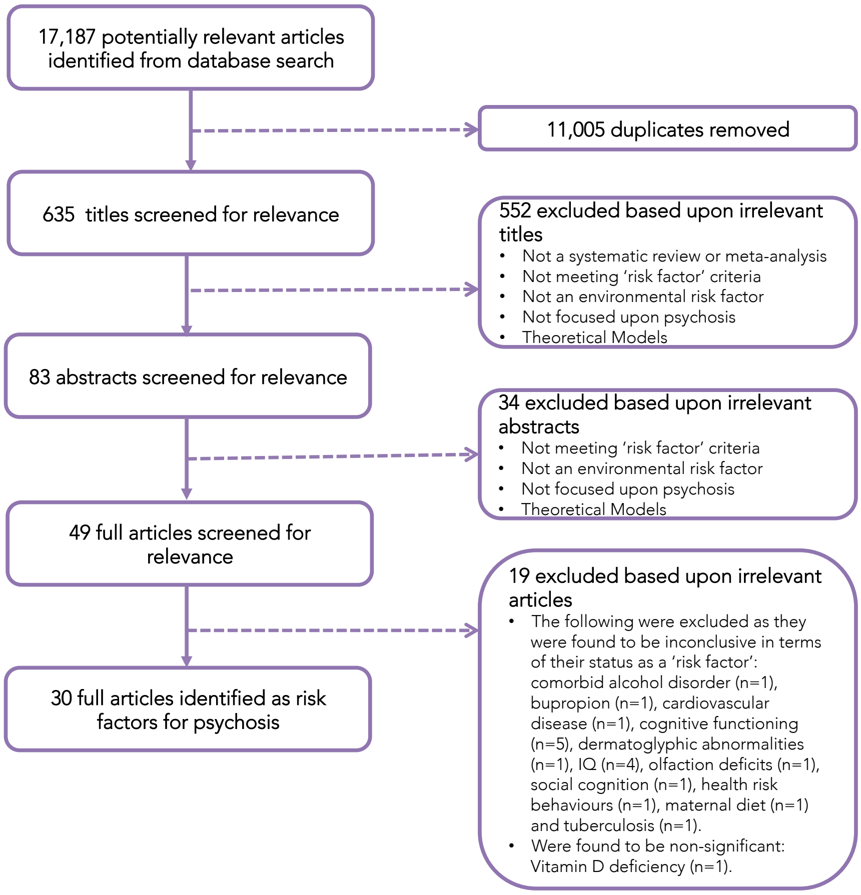

Flowchart of systematic search strategy.

Supplementary Materials 2 – OWLS Survey Data Cleaning Process

There were 3,477 survey responses in the raw data. 1,420 incomplete responses were initially removed, and the following cleaning process was then employed:

1. **De-duplication (177 responses removed):**
   a. Primary matching for duplicates was done based upon email address.
   b. Fuzzy matching on email address prefixes was performed to account for the same individual using different email providers (i.e. email domains).
   c. Possible matches were further verified by date of birth, gender and ethnicity comparison.

2. **Implausible Age Removal (19 responses removed):**
   a. Respondents who reported their age to be below 16 years (n=8), above 65 years (n=8) or reported a DOB that was implausible at the time of survey completion (n=3) were removed.

3. **Incongruent Sleep Timing Correction/Removal (3 responses removed):**
   a. 64 respondents either confused Bedtime and Lights Off.
   b. 78 respondents confused AM/PM timings for the PSQI and MCTQ.
   c. These entries were corrected if the time between bedtime and lights off was less than two hours. Responses with over an hour between bedtime and lights off were examined to see if the respondent was reporting a sleep problem. Each correction was performed in the context of the participants' responses: nothing was altered if the data looked implausible or the answers were incongruent – instead, these respondents were discarded (n=3).

4. **Incongruent PQ16 Distress Removal (46 responses removed):**
   a. 146 respondents reported distress for items on the PQ16 that they did not endorse as experiencing.
   b. Respondents who did this for more than one item on the PQ16 were discarded.

5. **Psychotic Disorder Removal (23 responses removed):**
   a. Respondents reporting a psychotic disorder were also excluded (n=23)

This left a total of 1,789 survey responses available for analysis.

Supplementary Materials 3 – Further Description of Statistical Analyses

Overview

For each PE dimension (as defined in **Supplementary Tables 2 & 3**), we built a multivariate logistic regression model, using the set of predictor variables (risk factors, demographics, sleep variables and psychopathology measures) to predict the binary response of whether any of the PQ16 questions contained in the PE dimension had been endorsed.

Both forward selection and backward elimination (standard model selection procedures) were used to propose candidate models. Beginning with a simple intercept-only model (with no predictor variables), forward selection iteratively adds the predictors offering maximal reduction to Akaike's Information Criterion (AIC), until no further reduction is possible. Backward elimination instead iteratively removes predictor variables from a complex model until no further reduction in AIC is possible.

Types of Output

The estimated regression coefficients from the resulting models were exponentially transformed into odds ratios, thus giving a measure of risk associated with each predictor variable. Further, for each survey respondent, we used the estimated regression coefficients to derive the estimated probability of endorsing at least one PQ16 question within the PE dimension. These predicted probabilities were compared to the known responses to gauge the quality of the models.

ROC curves were plotted, showing the true positive rate (sensitivity) against the false positive rate (1-specificity) for varying classification thresholds, and providing a visual representation of the models' ability to reliably discriminate between those endorsing and not endorsing at least one PQ16 item within each PE dimension. A model with high discrimination ability will simultaneously have a high true positive rate and a low false positive rate (i.e. high sensitivity and specificity), leading to an ROC curve that approaches the top-left corner of the plot. A model with poor discrimination ability will lead to an ROC curve that approaches the 45-degree diagonal line (the 'line of no discrimination, which is equivalent to a coin-toss).

The area under the ROC curve (AUC) was also calculated. This area can be interpreted as the probability that a randomly chosen participant who endorsed the psychotic symptom and a randomly chosen participant without the psychotic symptom could be reliably distinguished based on their responses to the main effects in the model. A value of 1 corresponds to a model offering perfect discrimination, and 0.5 corresponds to a model with no discrimination ability.

Excluded Risk Factors

Migrant status (1st and 2nd generation), 22Q11.2 deletion syndrome, epilepsy, latitude, brain injury and brain infection were excluded from the set of potential predictor variables, as they were infrequently endorsed in our sample. Furthermore, the majority of this sample (69%) possesses high levels of education (already holding a bachelors, masters or PhD, or currently studying towards a tertiary qualification). As such, it was deemed that the level of education would simply act as a proxy for age as opposed to a descriptive variable of education level, and for this reason, it was excluded from the analysis.

Stress (from the DASS questionnaire) was very highly correlated with both anxiety ($r=0.74$) and depression ($r=0.71$), and was accordingly excluded from the analysis due to concerns of collinearity. Whilst still relatively high ($r=0.63$), the correlation between anxiety and depression was lower, and these were both included in the set of potential predictor variables.

Results

In addition to the ROC curves presented in the main body, the boxplots below provide a visualisation of the models' discrimination abilities – i.e. how effective each model is at correctly predicting whether an individual endorsed each PE dimension - for the individuals in the randomly selected training dataset (consisting of 70% of the data).

The logistic regression model for negative symptoms demonstrates excellent discrimination ability between participants who did/did not endorse negative symptoms, with good separation between the boxes. Similarly, the models for paranoia, bizarre ideas, perceptual abnormalities and cognitive disorganisation show clearly distinguishable differences in the predicted probabilities for those who did/did not endorse the outcome. The model for delusional mood had the greatest overlap, but still separated the groups reasonably well.

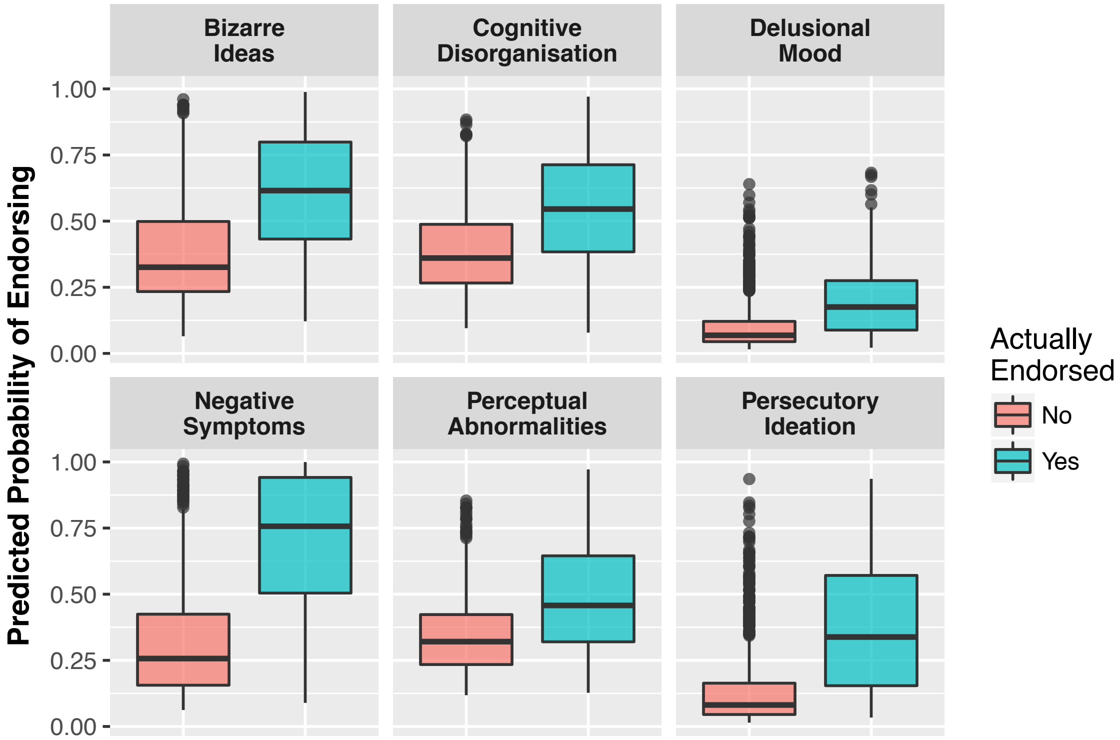

Figure: Boxplots of predicted probabilities from each of the six logistic regression models for survey respondents in the training dataset. The predicted probabilities for those respondents who endorsed each subcategory are compared to those who did not.

Supplementary Table 1: Overview of the risk factors identified based on a systematic review of the literature

Risk Factor	Reference	Disorder	Type	K	N	Het.	Effect Size	CI
**Family History**	Rasic et al., 2013	BP, SZ, D	Meta	33	7021	S	2.7	2.2-3.4
	Van Snellenberg &Canada 2009	SZ, BP	Meta	66	47,777		8.38	NA
**Ethnicity**	Kirkbridge et al., 2012	SZ	Meta	5	1,423	S	2.4 - 5.9	3.4-9.2
**Obstetric Complications**	Cannon et al., 2002	SZ	Meta	8	1,923	NS	1.4 - 7.8	NA
**Parenting**	De Sousa et al., 2013	P	Meta	20	1,753	S	5.8	3.9 –8.5
**Child Abuse**	Matheson et al., 2013	SZ	Meta	25	1,681	S	3.6	2.1–6.2
	Varese et al.,2012	P	Meta	36	81,262	S	2.8	2.3–3.3
	Norman et al., 2012	D, SA, A, ED, SZ	Meta	388	1,186,074	S	1.4-2.3	1.2-5.7
	Chen et al., 2010	A, D, ED, SZ, BP	Meta	71	3,162,318	S	1.4-2.3	2.4 -3.9
**Migration**	Borque et al., 2011	SZ	Meta	21	67,551	S	2.1 - 2.3	N/A
	Cantor-Graae &Selten 2005	SZ	Meta	315	31,045	S	RR=2.7	2.3-3.2
**Cannabis Use**	Moore et al., 2007	P, A	Meta	33	61,485	NS	1.4-2.9	1.11-7.57
	Semple et al., 2011	P	Meta	11	113,802	NS	2.9	2.4-3.6
**Traumatic Life Events**	Beards et al., 2013	P	Meta	16	2,465	S	3.2	2.2–4.8
	Trevillion et al., 2012	D, A, ED, PD, BD, P	Meta	41	69,893	S	2.8 - 8.1	4.5–12.0
**Bullying**	Van Dam et al., 2012	P	Meta	14	2,883	N/A	2.3	1.5-3.4
**Brain Injury**	Molloy et al., 2011	SZ	Meta	9	169,925	S	1.65	1.2-2.3
**Axis I Diagnosis**	Achim et al., 2011	SZ	Meta	52	10,731	S	Varied	Varied
**Gender**	Aleman et al., 2003	SZ	Meta	38	N/A	S	1.42	1.3-1.6
**Latitude and Climate**	Cheng et al., 2008	SZ	Meta	9	3,365,722	S	1.51	1.3-1.8
**Paternal Age**	Miller et al., 2011	SZ	Meta	12	195.092	S	RR=1.66	1.5-1.9
**Prenatal Infections**	Khandaker et al., 2013	SZ	SR	21	1,217,472	S	N/A	N/A
**Season of Birth**	McGrath et al., 1999	SZ	Meta	12	20,017	S	1.04	0.9-1.1
	Davies et al., 2003	SZ	Meta	8	86,732,003	S	1.07	1.05-1.1
**Social Withdrawal**	Matheson et al., 2013	SZ	Meta	6	3,828	S	SMD=1.04	0.3-1.8
**Urbanicity**	Vassos et al., 2012	SZ	Meta	4	46,820	S	2.39	1.6-3.5
**Childhood Viral Infection**	Khandaker et al., 2012	SZ	SR	21	7	NS	RR=1.7	1.1-2.6
**22q.1 Deletion Syndrome**	Armando et al., 2013	SZ	SR	9	13,026	N/A	N/A	N/A
**Epilepsy**	Clancy et al.,2014	P	Meta	58	N/A	S	7.8	2.8-21.8

Note. Disorders are abbreviated as follows: SZ=Schizophrenia; BP=Bipolar Disorder; D=Depression; P=Psychosis; ED=Eating Disorders; A=Autism; PD=Personality Disorder; SA= Substance Abuse. The type of article is abbreviated to either Meta (meta-analysis) or SR (Systematic Review). Heterogeneity is abbreviated to either S (for significant) or NS (for not significant). Effect sizes are measured in odds ratios unless otherwise specified, as are confidence intervals. K refers to the number of studies included in the review, and N refers to the total number of participants included in the review.

Supplementary Table 2: Risk Factors, Questionnaires and Sociodemographic Variables Employed in the OWLS survey

Item assessed	Assessment method
**Psychosis symptomatology**	Prodromal Questionnaire-16 (PQ16; Ising *et al.*, 2012).
**Subjective sleep quality**	Pittsburgh Sleep Quality Index (PSQI; Buysse *et al.*, 1989).
**Insomnia**	Short version of Sleep Condition Indicator (SCI-2; Espie *et al.*, 2014).
**Social jetlag & mid-sleep time on free days**	Munich Chronotype Questionnaire (MCTQ; Roenneberg *et al.*, 2003).
**Axis I disorder symptomatology**	Depression Anxiety Stress Scale-21 (DASS-21; Henry *et al.*, 2005)
**Brain infection**	'Have you ever suffered from encephalitis, meningitis or an infection of the brain before the age of 16 which required hospitalisation for more than one day?
**Brain injury**	'Have you ever suffered from a brain injury that required hospitalisation for more than one day?'
**Cannabis use**	A series of questions relating to current frequency of cannabis use, highest frequency of use and duration of most frequent use.
**Childhood abuse**	Questions on frequency of physical, sexual, psychological and emotional abuse before the age of 16 as used by Cuijpers *et al* (2011)
**Childhood bullying**	'When you were at school were you the victim of frequent bullying?'
**Childhood social withdrawal**	Four measures of social withdrawal as a child taken from items 42, 65, 88 & 111 from the Child Behavioural Checklist (CBCL; Achenbach et al., 1983) and edited to make appropriate for retrospective report
**Diagnosis of a non-psychotic psychiatric disorder**	Diagnoses and treatment used if applicable
**Ethnicity**	Ethnicity question taken from the national census
**Family history of psychiatric disorders**	Number of blood relatives diagnosed with a mental health disorder, their diagnosis, and treatment if applicable
**First or second generation migrant**	Participants country of birth, participants country of permanent residence, parents country of birth
**Gender**	'What is your sex/gender?'
**Help seeking behaviour**	'Have you ever sought help for any of the above [psychosis-like] experiences?' (including counselling, GPs)
**Lack of need for sleep**	'During the past month, have you had much less sleep than usual, found you didn't really miss it and did this cause a problem?'. Adaptation of item 4 of the Mood Disorder Questionnaire (MDQ, Hirschfeld *et al.*, 2000).
**Latitude**	Participants clicked which region on a world map where they have lived the longest between the ages of 0-18 years
**Obstetric complications**	List of obstetric complications given. Participants were asked to ring their mother if willing.
**Paternal age**	'How old was your father when you were conceived?'
**Season of birth**	'What is your date of birth?'

**Traumatic experiences**	Life Threatening Experiences (LTE) scale with distress levels added (Brugha *et al.*, 1985)
**Urbanicity**	'Is where you have lived the longest between the ages of 0-18 years a densely populated crowded city?' Examples given.
**22q11.2 deletion syndrome diagnosis**	Diagnosis present: Y/N
**Epilepsy**	Do you suffer from epilepsy (Y/N)
**Level of Education**	What is the highest level of education you have ever completed?<br>Are you currently working towards a higher level of education? Y/N<br>If Y: What level of education are you currently working towards?

Supplementary Table 3: Summary of variables measured by the OWLS survey, with descriptions of the data/type of measurement resulting from the survey.

Item assessed	Method of Measurement
**Outcome Measure**	
PQ16 (psychotic experiences)	a. Count score (of 16 items) measured continuously<br>b. A score above 5<br>c. A score above 5 with associated distress and help seeking behaviour
**Sleep Predictor Variables**	
PSQI (sleep quality)	Measured continuously (a score of 5 or above indicates poor quality sleep)
SCI-2 (insomnia)	Measured continuously (the lower the score the worse the sleep complaint)
MCTQ (Chronotype)	a. Chronotype category score (0=neutral; 1=morning type; 2=late type)<br>b. MSFsc – measured in time continuously
MDQ (decreased need for sleep)	Measured categorically (0='No' to 4='Yes – Serious Problem')
**Psychiatric Symptomatology Predictor Variables**	
DASS 21 (Dep/Anx/Stress)	Measured categorically for depression, stress and anxiety (0='Never', 1='Sometimes', 2='Often', 3='Almost Always').
**Risk Factor Predictor Variables**	
Brain infection	Binary (Y/N)
Brain injury	Binary (Y/N)
Cannabis use -ever used	Binary (Y/N) - Have you ever taken cannabis?
Cannabis use - now	Measured categorically: in the past three months, how often have you used cannabis? (0='Never'; 4='Daily or Almost Daily').
Childhood abuse	Categorical frequency of each physical, sexual, psychological and emotional abuse before the age of 16 (0='Never'; 5='Very Often').
Childhood bullying	Binary (Y/N)
CBCL (social withdrawal)	Sum of four questions with three categories (0='Never'; 1='Sometimes'; 2= 'Often').
Diagnosis of non-psychotic disorder	Binary – presence of any diagnosis (Y/N)
Ethnicity	Binary – A score of one is given when a respondent identifies as 'non-white'
Family history - First Degree (SZ/BP)	Binary (Y/N) - A First Degree Relative with schizophrenia/bipolar
Family history - First Degree (Other)	Binary (Y/N) - A First Degree Relative with other psychiatric diagnosis
First or second generation migrant	Binary – score given if respondent was a first/second generation migrant from a less developed or developing country to a first world country
Gender	Binary – if respondent endorses being 'male', a score of 1 is given.
Latitude	Binary – A score of 1 was given to participants who endorsed regions 1 or 2 indicating the most northern regions on a world map
Obstetric complications	Binary – If respondent endorses one or more obstetric complication a score of 1 is given.
Paternal age	Binary – If respondent endorses their father to be 50 or over at age of conception OR below 24 or below, a score of 1 is given.
Season of birth	Binary – if respondent has a birthday during the winter/spring months, a score of 1 is given.
Traumatic experiences	Measured continuously as count score for the number of traumas (out of a total 12) experienced.
Traumatic experiences - distress	Total of the distress associated with each trauma experienced (0= "Not Stressful", 1= "Slightly Stressful", 2= "Moderately Stressful", 3= "Very Stressful")
Urbanicity	Binary (Y/N)
22q11.2 deletion syndrome	Binary (Y/N)
Epilepsy	Binary (Y/N)
**Covariates**	

Level of education	This is categorised into 4 groups: low (pre A-level), medium (A-level, further college of education), high (bachelors), very high (MA/PhD).
Studying towards	Binary (Y/N)
Age	Measured continuously in years

Supplementary Table 4: PE Dimensions

Item Number	Item on PQ 16	Item on PQ92	Category on PQ92	Positive or Negative	PE Dimension for Modelling
1	I feel uninterested in the things I used to enjoy.	89	Avolition	Negative	Negative Symptoms
2	I often seem to live through events exactly as they happened before (déjà vu.)	8	Perplexity	Positive	Cognitive Disorganisation
3	I sometimes smell or taste things that other people can't smell or taste.	9	Olfactory	Positive	Perceptual Abnormalities
4	I often hear unusual sounds like banging, clicking, hissing, clapping or ringing in my ears.	18	Auditory	Positive	Perceptual Abnormalities
5	I have been confused at times whether something I experienced was real or imaginary.	57	Perplexity	Positive	Cognitive Disorganisation
6	When I look at a person, or look at myself in a mirror, I have seen the face change right before my eyes.	4	Visual	Positive	Cognitive Disorganisation
7	I get extremely anxious when meeting people for the first time.	80	Social Anxiety	Negative	Negative Symptoms
8	I have seen things that other people apparently can't see.	84	Visual	Positive	Perceptual Abnormalities
9	My thoughts are sometimes so strong that I can almost hear them.	65	General	Positive	Bizarre Ideas
10	I sometimes see special meanings in advertisements, shop windows, or in the way things are arranged around me.	67	Reference	Positive	Bizarre Ideas
11	Sometimes I have felt that I'm not in control of my own ideas or thoughts.	27	Telepathy	Positive	Bizarre Ideas
12	Sometimes I feel suddenly distracted by distant sounds that I am not normally aware of.	50	Auditory	Positive	Perceptual Abnormalities
13	I have heard things other people can't hear like voices of people whispering or talking.	13	Auditory	Positive	Perceptual Abnormalities
14	I often feel that others have it in for me.	25	Paranoid	Positive	Persecutory Ideation
15	I have had the sense that some person or force is around me, even though I could not see anyone.	52	General	Positive	Delusional Mood
16	I feel that parts of my body have changed in some way, or that parts of my body are working differently than before.	64	Somatic	Positive	Perceptual Abnormalities

Above are the categorisations of the Prodromal Questionnaire (92-Item Version; Loewy, Bearden, Johnson, Raine & Cannon 2005) detailed by Therman (2014) in "Mapping the uncanny: Assessing dimensions of psychotic-like experiences for clinical utility". Therman (2014) identifies 26 subcategories of experience, 11 of which are present in the PQ 16. The document which specifically maps the 26 categories to the 92 items of the questionnaire is not published (to the author's knowledge) and the authors were contacted directly for them, they can be forwarded on request. This provides 11 categories for the PQ16 which would have made modelling unfeasible due to issues with power and the some of the category labels (ex: general) were not informative. As a result we collated the olfactory, 3 x auditory, 2 x visual and somatic items into a group of perceptual abnormalities. This is based on Section 1.3 of the Comprehensive Assessment of At Risk Mental State (CAARMS) by Yung et al., (2005). Avolition and social anxiety are put together to form the negative symptoms category as they are in the paper by Isling et al., (2012) which

describes the validity of the PQ-16. The two items of perplexity and one of the items under the visual category "When I look at a person, or look at myself in a mirror, I have seen the face change right before my eyes.", were added to the cognitive disorganisation category. This is because this item is considered a core feature of depersonalisation (Carlson & Putnam, 1993) and it was important to balance groups where possible to allow for greater prediction accuracy of the models. Bizarre ideas is comprised of a general item, two ideas of reference items and an item on telepathy. This is in line with the categories set out by Comprehensive Assessment of At Risk Mental State (CAARMS) by Yung et al., (2005). This left two items one from the general category which is labelled as delusional mood (again based on the CAARMS) and another which measured paranoia. These were given their own categories labelled "delusional mood" and "persecutory ideation". The categories were discussed and agreed upon by the research team (including a consultant psychiatrist and psychologist) prior to any modelling taking place.

Supplementary Table 5: A cross-sectional examination of risk factors across the number of PE (n=1789).

Prediction	Risk Factor	Variable	x^2	df	p	Adj. p
**Psychopathological**	Diagnoses	All Diagnoses	220·2	3	<·0001	<·0001
**Continuity**	Psychometrics	Sleep Quality	218·6	3	<·0001	<·0001
		Insomnia	138·9	3	<·0001	<·0001
**Need for Care**	Treatment	Counselling	211·1	3	<·0001	<·0001
		Medication	181·4	3	<·0001	<·0001
		Hospitalisation	60·4	3	<·0001	<·0001
**Demographics***	Urbanicity		3·9	3	0·27	0·29
	Ethnicity	Non-white	8·2	3	0·043	0·053
	SOB		1·1	3	0·771	0·771
	Migrant		3·7	3	0·300	0·314
	Paternal Age		7·0	3	0·072	0·083
**Aetiological Risks**	Family History**		37·1	3	<·0001	<·0001
	Obstetric C.		18·7	3	0.003	0.004
	Bullying		64·3	3	<·0001	<·0001
	Social Withdrawal		65·9	3	<·0001	<·0001
	Child Abuse	Physical	75·9	3	<·0001	<·0001
		Sexual	39·1	3	<·0001	<·0001
		Psychological	119·9	3	<·0001	<·0001
		Emotional	147·2	3	<·0001	<·0001
	Cannabis Use	Ever	19·2	3	<·0001	<·0001
		Now	19·9	3	<·0001	<·0001
	Trauma	Ever	38·2	3	<·0001	<·0001

As the sample was uniformly highly educated, we did not investigate educational differences between groups.

*There were not a sufficient number of endorsements of 22Q11.2 syndrome, latitude, epilepsy, brain injury, and brain infection to examine these proportionally across groups.

**Family history of schizophrenia/bipolar disorder and other disorders needed to be combined, as it was otherwise underpowered to detect differences. Only first-degree relatives with a mental health disorder were considered.

Supplementary Table 6: Classification success rate and the Area Under the Curve for the training and the test data for each PE dimension.

		Classification Success Rate		AUC (Area Under the Curve)	
	**Classification Threshold**	**Training Data (70%)**	**Test Data (30%)**	**Training Data (70%)**	**Test Data (30%)**
Negative Symptoms	0·44	78·3	78·8	0·86	0·87
Perceptual Abnormalities	0·39	66·5	67·6	0·71	0·70
Bizarre Ideas	0·46	71·3	67·2	0·78	0·73
Persecutory Ideation	0·20	78·3	76·2	0·82	0·82
Delusional Mood	0·10	68·6	69·1	0·75	0·73
Cognitive Disorganisation	0·46	68·3	68·3	0·73	0·74

The classification success rate is the percentage of respondents correctly classified as endorsing/not endorsing the PE dimension, based on comparing their predicted probability of endorsement to the threshold provided (determined from the corresponding ROC curve). The AUC can be interpreted as the probability, based on their responses to the main effects in the model, of distinguishing a randomly chosen respondent who endorsed the PE dimension from a randomly chosen participant who did not.
